# Wrist flexion and extension torques measured by highly sensitive dynamometer in healthy subjects from 5 to 80 years

**DOI:** 10.1186/s12891-015-0458-9

**Published:** 2015-01-31

**Authors:** Valérie Decostre, Aurélie Canal, Gwenn Ollivier, Isabelle Ledoux, Amélie Moraux, Valérie Doppler, Christine Anne Mary Payan, Jean-Yves Hogrel

**Affiliations:** Institut de Myologie, GH Pitié-Salpêtrière, Paris, France; Département de Pharmacologie clinique, GH Pitié-Salpêtrière, Paris, France

**Keywords:** Wrist muscle strength, Norms, Predictive model, Outcome measures

## Abstract

**Background:**

Wrist movements become impaired with disease progression in various neuromuscular disorders. With the development of new therapies, thorough measurement of muscle strength is crucial to document natural disease progression and to assess treatment efficacy. We developed a new dynamometer enabling wrist flexion and extension torque measurement with high sensitivity. The aims of the present study were to collect norms for healthy children and adults, to compute predictive equations, to assess the reliability of the measurements and to test the feasibility of using the device in patients with a neuromuscular disease.

**Methods:**

The peak isometric torque of wrist flexion and extension was measured with the MyoWrist dynamometer in 345 healthy subjects aged between 5 and 80 years old and in 9 patients with limb girdle muscle dystrophy type 2 C (LGMD2C) aged between 16 and 38 years old.

**Results:**

Predictive equations are proposed for the wrist flexion and extension strength in children and adults. Intra-rater and inter-rater reliability was good with ICCs higher than 0.9 for both wrist flexion and extension. However, retest values were significantly higher by 4% than test results. The dynamometer was applied with no difficulty to patients with LGMD2C and was sensitive enough to detect strength as weak as 0.82 N.m. From our models, we quantified the mean strength of wrist extension in LGMD2C patients to 39 ± 17% of their predicted values.

**Conclusions:**

The MyoWrist dynamometer provides reliable and sensitive measurement of both wrist flexion and extension torques. However, a training session is recommended before starting a study as a small but significant learning effect was observed. Strength deficit can be quantified from predictive equations that were computed from norms of healthy children and adults.

## Background

With new therapies emerging for myopathies [1,2], the need for reliable outcome measures of the upper limb has become crucial, particularly for clinical trials including non-ambulant patients. Change in wrist strength and function may impact upper limb abilities and therefore, the quality of life of these patients.

Wrist flexion and extension strength have been previously assessed using different methods: Manual muscle testing (MMT) [3-5], hand-held dynamometry (HHD) [6,7], isokinetic dynamometry [8-10] and home-made dynamometers [11-13]. Advantages and disadvantages of these various methods have previously been discussed [14]. None of these methods is reliable and highly sensitive over a large range of strengths as well as being flexible for different upper limb dimensions or deformities (e.g. contractures).

We developed a highly sensitive device, called the MyoWrist, specifically designed for the assessment of both wrist flexion and extension torque with the same upper limb positioning in children and adults with the possibility of wrist angle adjustment for subjects with contractures. Quantifying the strength of patients relative to a healthy population is useful to assess a deficit or identify improvements.

The aims of the present study were to establish norms and predictive equations for both wrist flexion and extension strength measured with the MyoWrist dynamometer from a population of healthy children and adults, to assess the repeatability of the measurements and to test the feasibility and consistency of the method in a group of patients with a neuromuscular disorder.

## Methods

### Subjects

This is a cross-sectional study which took place in the Institute of Myology in Paris between November 2006 and December 2008. Healthy male and female subjects aged between 5 and 80 years old were recruited by advertisements in newspapers, websites, posters and open access animation for the French Telethon. The socio economic status, blue or white collar worker and urban or rural environment of the subjects were not recorded, but since all the measurements took place in Paris, the urban middle class white collar profile is probably over-represented. Therefore, the results presented in this study are not representative of the French population and the terms “norms” or “normative data” used here only refer to our tested sample. Exclusion criteria comprised of history of injury or disease involving the upper limb in the past two years, pain or discomfort that could affect upper limb performance or sport practiced at a national level. The so-called MyoTools protocol was approved by the Local Ethics Committee (CPP-Ile de France VI; La Pitié-Salpêtrière).

Data of patients with Limb Girdle Muscular Dystrophy type C (LGMD2C) caused by γ-sarcoglycan deficiency were also analysed in order to demonstrate the feasibility and sensitivity of using the dynamometer in subjects with strength weakness. Limb girdle muscle dystrophy was chosen for proof of concept of the use of the device in patients with neuromuscular disease as the disease is characterized by progressive proximal muscle weakness [15]. Strength of distal muscles was therefore expected to remain stable in a one month period for test – retest measurements. The patient data were taken from the protocol Eudract no. 2006-005132-24, ISRCTN no. 22225367 and clinicalTrials.gov no. NCT01344798 approved by the Pitié-Salpêtrière Hospital ethics committee and were already published [16]. Each participant was informed about the experimental protocol and procedures before signing informed consent.

### Anthropometric measurements

Height, body mass, percentage of body fat mass measured by impedance metric scale (TBF-543, Tanita Corporation, Arlington Heights, Illinois, USA), forearm circumference (measured at the largest part of the forearm, usually located at the proximal ¼ forearm depending on morphology), hand circumference (perimeter at mid-hand at the level of main palmar creases) and hand length (distance between the palmar wrist crease and the distal extremity of the middle finger) were measured. Upper limb anthropometric measurements were performed to the nearest mm using a standard tape measure on subjects sitting with the supinated forearm supported on the thigh. The dominant side was defined as the hand used by the subject to write or draw with.

### Dynamometer description

**Figure 1:**
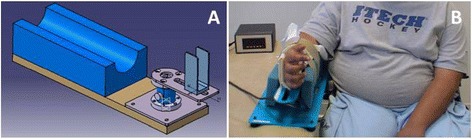
**MyoWrist design (A) and subject positioning into the device (B) for the measurement of wrist flexion and extension.** The patient photographed in this figure gave his specific written informed consent to have his image published.

A complete description of the MyoWrist dynamometer was previously published (Appendix 1 in [17]. A torque meter (DF30-25 Nm; Scaime; France) inserted into a homemade device measured the isometric torque of wrist flexion and extension. The torque sensor was fixed on an aluminium plate equipped with a foam cradle and two Velcro straps in order to firmly maintain the forearm within the cradle. Two vertical bars were positioned at the elbow level aiming to avoid lateral movements of the elbow. A U-shaped support was fixed over the torque meter to receive the hand metacarpi within a dense foam cradle. This support was adjustable with respect to the hand length and can be rotated to study the torque generated by the wrist extensors and flexors at different joint angle (Figure 1A). In the present study, the U-shaped support was positioned to align the wrist axis for flexion/extension with the torque meter axis and so that the subject’s wrist was at 0° of flexion and extension. With its light mass (2.9 kg) and small dimensions (54 × 20 × 20 cm), the wrist dynamometer is easily transportable.

Calibration of the sensor and its electronic connections was performed according to ISO 9001 and 17025 norms of quality assurance and were certified by the manufacturer. The nominal torque of the transducer was 25 N.m, its precision was 0.05 N.m and its sensitivity was 0.0025 N.m. Signal from the transducer was conditioned by an electronic board (Scaime, type CPJ). A Scaime type PAX S programmable indicator displayed on a digital screen either the real time torque or the maximal value for wrist flexion or extension. A BNC output allows the analogue torque signal to be displayed on a computer screen or recorded for further analyses.

### Experimental procedure

The healthy subjects were tested sitting on a chair with the trunk in an upright position, while LGMD2C patients were assessed in their wheelchair. The forearm was positioned horizontally within the cradle of the device placed on a height adjustable plinth. The height of the plinth was adjusted so that the upper limb was positioned at 30° ± 10° shoulder abduction and at 30° ± 10° shoulder flexion. The elbow angle was set at 60° ± 10° of flexion and the forearm was placed in neutral pro-supination position in the device. The feet were flat on the floor or on the footrests of the wheelchair. The contralateral hand was kept on the thigh (Figure 1B).

Subjects were instructed to alternately produce their maximal wrist extension and flexion strength. A trial consisted of a 2–4 s maximal voluntary contraction (MVC). Each trial was followed by a 30s rest period. Two trials were first recorded. If there was ≤10% difference between both trials, no additional measurement was required. Otherwise, supplementary trials were proposed until two MVCs reproducible within 10% were obtained within a maximum of 5 trials. The maximal reproducible value within 10% was retained for the analyses. The first side (right or left) and direction (extension or flexion) tested were randomly assigned. The subjects were verbally encouraged to produce their best performance.

Re-test was performed with healthy subjects who agreed to participate in a second session between 1 hour and 30 days after the first session. Three evaluators participated in the measurements for reliability assessment. Unless impossible, the same evaluator performed the test and retest measurements.

### Statistical analyses

Statistical analyses were performed with the SPSS version 19 software. Results are presented as means ± standard deviation and the limit of significance was set at p < 0.05.

In order to determine whether norms should be specified for each upper limb side or not, significant differences between dominant and non-dominant sides were analysed by a paired *t*-test in right- and left-handers.

Coefficients of predictive equations were determined separately on children younger than 18 years old and adults over 18 years old. In both groups, a stepwise linear regression selected the best predicting variables among sex, age, body mass, height, body mass index, fat mass percentage, forearm circumference, hand circumference and hand length for the natural logarithm of wrist flexion and extension torque.

Repeatability of the torque measurements was assessed comparing test and retest values of wrist flexion and extension with repeated measures ANOVA. In addition, the intra-class correlation coefficient (ICC), the standard error of measurement (SEM) and the coefficient of variation (CVar) were also determined. ICC was computed as single measures with a two-way random effect model (absolute agreement). Cvar was computed for all subjects regardless of whether they were assessed by different evaluators or not. The smallest detectable difference (SDD) was computed as previously described [18].

The repeatability of wrist extension torque measurements in patients with LGMD2C was assessed by a Wilcoxon test. Torque was then expressed in percent of predicted values in order to demonstrate the feasibility of measurements with the device in a small sample of weak subjects with neuromuscular disease.

## Results

### Subjects

This study enrolled 345 healthy subjects among which 57 were children under 18 years old (28 boys and 29 girls) and 288 were adults (119 men and 169 women) whose characteristics are given in Table 1. The left side was not tested in two subjects (a 32 and a 34 year-old women) and wrist flexion of the right side was not measured in a 54 year-old woman due to reported discomfort.

**Table 1:** **Subjects’ characteristics presented as mean (SD)**

**Age (year)**	**Sex**	**n**	**Height (cm)**	**Body mass (kg)**	**Body fat mass (%)**	**Forearm circumference (mm)**	**Hand circumference (mm)**	**Hand length (mm)**	**Wrist flexion (N.m)**		**Wrist extension (N.m)**	
									**Left**	**Right**	**Left**	**Right**
5-9	F	12	127.8 (10.4)	28.3 (7.8)	24.0 (4.5)	18.5 (1.7)	15.5 (1.0)	14.3 (1.2)	3.6 (0.7)	3.4 (1.0)	2.4 (1.1)	2.5 (1.0)
	M	14	118.5 (8.4)	22.8 (4.8)	13.9 (4.9)	17.2 (1.3)	15.2 (0.7)	13.2 (0.8)	3.2 (1.2)	2.9 (0.8)	1.7 (0.7)	2.0 (0.7)
10-14	F	9	154.1 (11.2)	44.1 (11.5)	23.6 (3.9)	20.9 (2.2)	17.5 (0.8)	16.6 (0.9)	5.9 (1.9)	5.9 (1.6)	4.1 (1.5)	4.7 (1.8)
	M	11	155.5 (10.4)	44.8 (9.9)	16.7 (4.5)	21.3 (1.8)	18.5 (1.0)	17.1 (1.0)	7.4 (3.2)	7.2 (2.8)	5.0 (1.6)	4.9 (1.3)
15-19	F	15	164.6 (5.0)	59.4 (11.4)	26.3 (6.1)	23.5 (2.0)	18.5 (0.9)	17.6 (0.9)	8.1 (2.3)	8.2 (2.7)	6.5 (1.1)	6.6 (1.6)
	M	10	182.0 (6.7)	74.9 (15.8)	15.4 (7.2)	26.4 (1.9)	21.3 (1.2)	19.9 (1.1)	14.0 (2.8)	13.8 (2.8)	9.6 (2.5)	10.1 (3.2)
20-29	F	32	167.2 (6.7)	64.4 (15.9)	29.6 (6.8)	23.7 (2.3)	18.9 (0.9)	17.9 (0.9)	8.2 (1.8)	8.2 (1.9)	6.0 (1.4)	6.5 (1.6)
	M	27	177.8 (4.9)	74.9 (10.3)	17.3 (5.7)	26.8 (1.6)	21.4 (0.8)	19.6 (0.7)	14.2 (3.2)	14.5 (2.8)	10.7 (2.4)	11.4 (2.1)
30-39	F	31	164.5 (5.8)	62.8 (10.1)	28.5 (7.9)	23.8 (2.1)	18.9 (1.7)	17.8 (1.0)	8.0 (1.9)	8.4 (1.8)	6.0 (1.8)	6.5 (2.1)
	M	32	176.5 (6.6)	76.4 (12.9)	18.7 (7.2)	26.5 (2.1)	21.5 (0.9)	19.3 (0.9)	13.3 (2.9)	13.4 (2.9)	9.1 (2.4)	10.2 (2.4)
40-49	F	32	163.8 (5.0)	62.4 (8.9)	28.5 (8.3)	23.4 (1.7)	19.0 (0.7)	17.7 (0.8)	8.3 (1.6)	8.0 (1.5)	5.4 (1.3)	6.4 (1.6)
	M	26	176.4 (6.1)	77.3 (12.9)	17.9 (5.5)	27.3 (2.0)	22.2 (1.0)	19.6 (1.0)	14.1 (2.9)	13.8 (2.8)	10.6 (2.5)	11.5 (2.4)
50-59	F	29	162.2 (6.1)	63.9 (10.7)	29.8 (7.6)	23.9 (1.8)	19.5 (0.8)	17.9 (0.8)	8.1 (1.7)	7.5 (1.4)	5.4 (1.1)	5.8 (0.9)
	M	11	178.3 (7.4)	78.8 (10.2)	19.0 (4.6)	27.1 (1.4)	22.3 (0.5)	20.0 (1.1)	13.6 (2.8)	13.9 (1.9)	9.7 (2.0)	10.6 (2.2)
60-69	F	21	160.2 (7.3)	62.8 (10.6)	29.8 (6.8)	23.2 (1.6)	19.4 (0.8)	17.8 (1.0)	7.5 (4.5)	6.8 (4.3)	5.6 (4.7)	5.7 (4.7)
	M	11	172.7 (6.8)	84.7 (13.0)	24.2 (5.5)	27.5 (1.9)	22.1 (1.0)	19.2 (1.0)	12.1 (2.9)	12.2 (3.0)	7.9 (2.0)	8.4 (2.3)
70-79	F	17	161.3 (5.0)	62.8 (8.1)	28.8 (7.8)	23.1 (1.5)	19.7 (0.7)	18.3 (0.7)	7.2 (1.6)	6.6 (1.3)	4.2 (1.3)	4.8 (1.5)
	M	5	173.2 (5.0)	84.3 (13.1)	22.8 (6.2)	27.6 (1.2)	21.9 (0.5)	19.3 (0.3)	13.5 (3.3)	11.8 (1.6)	8.2 (1.5)	8.3 (2.5)

Forearm, hand circumference and hand length are presented as a mean of the left and right side.

For subjects with a thin hand, additional foam had to be placed in the U-shaped support of the dynamometer to perfectly fix the hand within the device. Although the hand was firmly maintained, no subject complained of pain.

Eighty seven per cent of the subjects were right handed and 9.5% were left handed. Three and a half percent of the subjects were ambidextrous but wrote with their right hand and were analysed as right-handers. The measurement took approximately 10 minutes per side.

### Normative data

Torques on the dominant and on the non-dominant sides were compared to determine whether norms should be specified for each upper limb side or not. The wrist flexion torque was significantly stronger on the dominant side than on the non-dominant side in left-handers but not in right-handers, while wrist extension torque was significantly higher on the dominant side than on the non-dominant side in right-handers but not in left-handers (Table 2). Because the dominant side was not systematically the strongest side for each function in left- and right-handers, further analyses were performed without taking into account hand dominance and results are presented for each side.

**Table 2:** **Differences in torque values between dominant and non-dominant sides**

	**Wrist flexion**		**Wrist extension**	
	**Right handers**	**Left handers**	**Right handers**	**Left handers**
Mean (SD) dominant – non dominant side difference (N.m)	−0.11 (1.60)	0.58 (1.58)	0.59 (1.37)	−0.20 (1.31)
p-value (paired *t*-test)	0.23	<0.05	<0.001	0.40
Number of subjects	310	32	310	32

### Predictive model

Height and forearm circumference were the main predictors of wrist flexion and extension torque in healthy children, while age, gender and forearm circumference were the main determinants in healthy adults.

Forearm circumference depended on height for healthy children (Pearson correlation coefficient (*r*_*P*_) = 0.890, n = 57, P < 0.01) and adults (*r*_*P*_ = 0.556, n = 288, P < 0.01) and may therefore be a redundant factor. Moreover, forearm circumference can be more affected than height by muscle disease. Therefore, we considered only height as predictor of wrist flexion and extension torque in children.

**Figure 2:**
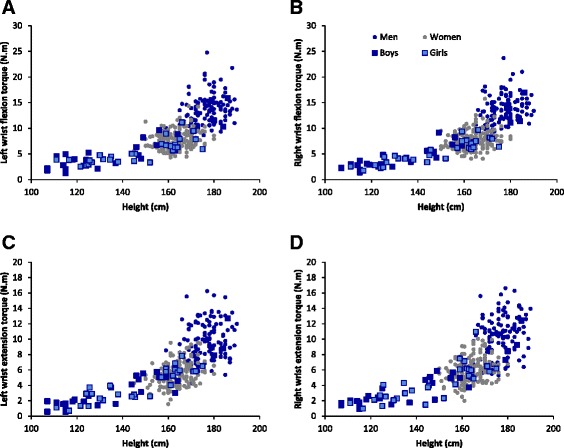
**Height dependence of torque for left wrist flexion (A) and extension (C) and for right wrist flexion (B) and extension (D).**

For the same reason, height was retained instead of forearm circumference in adults in addition to age and gender as main prediction factors of wrist flexion and extension torque. Figure 2 presents the height dependence of wrist flexion and extension torque in children and adults. Because the relationship appeared exponential, predictive equations based on a linear regression model were calculated for the natural logarithm of the torques. They are presented in Tables 3 and 4 for children and adults respectively. The adjusted R-squared was above 0.70 for children and above 0.55 for adults, indicating a better fit of the model for children than for adults.

**Table 3:** **Predictive models for children wrist flexion and extension torque**

**Torque natural logarithmic transformation (Ln (N.m))**	**Intercept**	**Height (cm)**	**Adjusted R** ^**2**^	**Model SD**	**n**
Ln left wrist flexion torque	−1.285***	0.02***	0.702	0.288	57
Ln right wrist flexion torque	−1.647***	0.022***	0.818	0.233	57
Ln left wrist extension torque	−2.804***	0.028***	0.757	0.345	57
Ln right wrist extension torque	−2.274***	0.025***	0.737	0.323	57

***P < 0.001.

**Table 4:** **Predictive models for adult wrist flexion and extension torque**

**Torque natural logarithmic transformation (Ln (N.m))**	**Intercept**	**Height (cm)**	**Age**	**Sex (0–1)** ^**#**^	**Adjusted R** ^**2**^	**Model SD**	**n**
Ln left wrist flexion	0.761*	0.008***	−0.002*	0.432***	0.632	0.214	286
Ln right wrist flexion	0.797*	0.008***	−0.003***	0.451***	0.680	0.205	287
Ln left wrist extension	0.553	0.008**	−0.005***	0.443***	0.584	0.254	286
Ln right wrist extension	0.439	0.009***	−0.005***	0.421***	0.578	0.254	288

^**#**^0 for women and 1 for men, *P < 0.05. **P < 0.01. ***P < 0.001.

### Reliability assessment

Seventy five of the 345 subjects (22%) performed a second assessment of wrist flexion and extension strength to assess the test-retest reliability of the measurements. Whenever possible, test and retest measurements were performed by the same evaluator (intra-rater reliability). However, 22 subjects (29%) could not be re-tested by the same evaluator (inter-rater reliability).

A repeated measures ANOVA (n = 150 pooling left and right sides) revealed a session effect (P < 0.01) with retest measurements being on average 4 ± 15% and 4 ± 18% higher than the test values for wrist flexion and extension, respectively. In addition, the analysis underscored a function effect (P < 0.001) with wrist flexion exceeding the extension strength by 40% on average. There was no interaction between session and function.

We examined whether the number of trials at the test session influenced the reliability at the retest session. Among the 75 subjects who performed the retest session, about 60%, 30%, 8% and 1% needed respectively 2, 3, 4 and 5 trials at the test session to meet the criteria of 10% reproducibility between 2 trials within a maximum of 5 trials. No relation or significant difference (ANOVA) could be evidenced between the test-retest reliability of the subjects who needed 2, 3, 4 or 5 trials at the test session.

**Figure 3:**
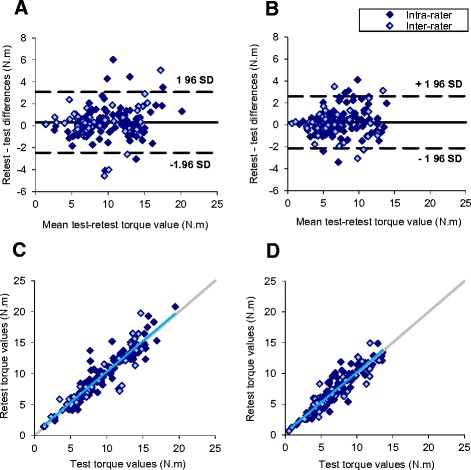
**Repeatability of wrist flexion (A and C) and extension (B and D) torque measurements assessed by Bland & Altman (upper panels) and correlation (lower panels) plots.** The blue line represents the linear regression of all data. The grey line illustrates the equivalence between test and retest.

Reliability properties of the wrist flexion and extension measurements are presented in Table 5. The limit of agreement between two successive measurements consists of the SDD that would indicate a real difference between the measurements with a 95% confidence for healthy subjects [18]. It reached 2.8 and 2.4 N.m for wrist flexion and extension measurements, respectively. Bland and Altman plot as well as the relationship between retest and test values for wrist flexion and extension are shown in Figure 3. The ICC for intra-rater (n = 106), inter-rater (n = 44) or all (n = 150) torque measurements was higher than 0.9 for both wrist flexion and extension, indicating a good reliability (Table 5).

**Table 5:** **Test-retest agreement and reliability**

	**Wrist flexion**	**Wrist extension**
Number of test-retest*	150	150
Mean retest-test difference (N.m)	0.3	0.2
SEM (N.m)	1.0	0.9
Relative SEM (%)	10	12
Limits of agreement (N.m)	2.8	2.4
Intra Class Correlation Coefficient	0.928	0.920

*Right and left side measurements of 75 subjects were pooled. SEM = Standard Error of Measurement.

### Feasibility in a small patient group

Torque measurement of wrist extension was measured in 9 patients with LGMD2C to illustrate the feasibility of the measurements in weak subjects. All patients were wheelchair-bound and right-handed. Patients’ age, gender and body mass are presented with height and dominant side data in Table 6. Mean test and retest measurements of wrist extension strength were previously published (Day −30 and Day 0 in Figure 1 of [16]). Thirty three percent of the retest measures could not be performed by the same evaluator as the test measurements. Retest values were significantly higher than the test measures with a mean torque difference of 12% (p < 0.05, n = 18 pooling right and left side measurements). However, the ICC of 0.879 between retest and test suggests a good correlation of both measurements.

**Table 6:** **LGMD2C patients’ characteristics**

**Patient number**	**Gender**	**Age (years)**	**Body mass (kg)**	**Height (cm)**	**Dominant side**
01	F	38	65	156	R
02	F	18	60	170	R
03	F	32	63	171	R
04	F	16	68	170	R
05	M	24	108	174	R
06	M	29	50	179	R
07	F	38	57	161	R
08	F	31	36	168	R
09	F	19	49	167	R

**Figure 4:**
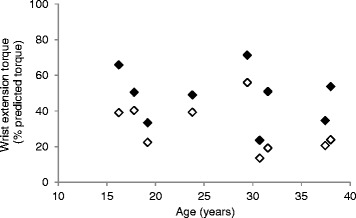
**Age dependence of right (empty symbols) and left (full symbols) wrist extension torque expressed in percent of predicted values for right-handed patients with LGMD2C.**

The relationship between age and wrist extension torque expressed in percent of the predicted value calculated from equations defined in Tables 3 and 4 is presented in Figure 4. Between 16 and 38 years of age, none of the patients developed a torque higher than 75% that of the predicted value and no age dependence of strength was observed. However, the dominant side systematically developed a weaker torque than the non-dominant side in these right-handed patients.

## Discussion

Wrist movements are involved in many tasks of daily life and can be impaired by neuromuscular disease. We have developed a dynamometer for the accurate and sensitive measurement of wrist flexion and extension torques. Norms were obtained from which predictive models were computed for children and adults. Torque measurements presented good repeatability between test and retest and could be performed in healthy and weak subjects with limb girdle muscle dystrophy.

### Feasibility of the measurements

The device is small enough to be easily transportable. It could be used to assess healthy children and adults as well as the weak patients with LGMD2C in the present study. Subjects with a thick hand were tightly bound in the U-shape support of the device but none complained of pain. The device was easily used in non-ambulant patients who remained in their wheelchair for the assessment. Both wrist flexion and extension torque can be measured with the upper limb remaining in the same position.

### Normative data

Norms reported in this study were obtained with wrist and forearm positioned at 0° flexion/extension and 0° pronation/supination, respectively, like in most previous studies. However, we chose a functional positioning for the elbow flexed at 60° and the shoulder flexed and abducted at 30° instead of the most frequently used positioning in 90° flexion of elbow and shoulder [19].

Isometric torque values measured with our MyoWrist dynamometer were in agreement with the wrist flexion and extension torques obtained in previous studies with the same device [17] or with other systems, at least for young men between 20 and 25 years old [20,21] or lower [10]. As previously observed for healthy subjects between 8 and 28 years old [17], no systematic relationship between hand dominance and wrist flexion and extension strength was demonstrated by our results.

### Predictive models

In the present study, height and forearm circumference were determined as the main predicting factors of wrist flexion and extension strength for children. Because forearm circumference was significantly correlated with height in healthy children, only height was considered as predictive factor of wrist flexion and extension strength. In adults, forearm circumference, age and gender appeared as the main wrist torque predictors. Because forearm circumference was also significantly correlated with height in adults, even if the Pearson correlation coefficient is smaller than for children, and because neuromuscular disease should impact height less than forearm circumference, we considered height instead of forearm circumference in addition to age and gender as predictive variables of wrist flexion and extension torque in adults.

In our previous studies, we already demonstrated the height dependence of hand grip, elbow flexion and extension, and knee flexion and extension strength in children between 5 and 17 years old [22].

A previous study proposed gender, body mass and age as predictive factors for wrist extensors force measured by HHD [6]. However, body mass is a multifactorial resultant while height is less influenced by factors such as regimen and muscle disease. Height is therefore expected to be a factor more adapted than body mass to predict wrist strength in patients with neuromuscular diseases.

The interest of the predictive models compared to the use of mean values as those presented in Table 1 for the prediction of the expected wrist torque of a subject is the precision and the number of subjects used for the prediction. As an example, the left wrist flexion torque of a 27-years old female 164 cm high can be precisely predicted from the model to 7.5308 N.m (=EXP (0.761 + 0.008* 164-0.002*27 + 0.432*0)), while Table 1 reports the 9% higher value of 8.2 N.m for females between 20 and 29 years old with a mean height of 167.2 cm. Moreover, in this example, the model prediction is based on an analysis of the results of 286 subjects, while the experimental mean value in Table 1 was obtained on 32 subjects. However, the fit of the model through the experimental torques is better for children than for adult data as the adjusted R^2^ is higher for the fit of children than for the fit of adult results (Tables 3 and 4). Using a model or the mean value from a normative table always remains a prediction with its imprecision. However, to determine the predicted torque value of a given subject, we favour calculation from the model with its fit imperfection rather than using the mean torque corresponding to an age category with a mean height as exampled in Table 1. First, this limits the effect of confounding variables such as stature, which is included in the model. Second, the model enables the expression in percentage of predicted value for the results of patients, allowing the quantification of a deficit and its evolution.

### Reliability assessments

Unlike a previous study with less participants (n = 30) [17], strength of wrist flexion and extension obtained with the MyoWrist device at the retest was only slightly but significantly higher than at the test session. A learning effect and less apprehension at the retest session may account for this effect. Even if the increase was small, variable and may have little clinical significance, we recommend familiarization session(s) with the MyoWrist before baseline measurements in clinical trials. The SEM and ICC values obtained in the present study confirm the results of a previous study using the same dynamometer [17].

As previously reported [10,21], we observed a higher torque for wrist flexion than for extension.

### Feasibility in a small patient group

Wrist extension torque measurements were applied in the 9 wheelchair bound patients with LGMD2C without practical difficulty. In case of contractures preventing the alignment of the hand with the forearm, the U shaped support of the device can be adjusted to the wrist angle. The MyoWrist dynamometer was previously used in adult patients with sporadic inclusion body myositis [23,24] or DMD [25] without measurement problems being reported. Nevertheless, in a larger group of young DMD patients between 10 and 27 years old, measurements with the MyoWrist set at zero degrees of wrist flexion/extension could be performed only in half of the 30 patients because of upper limb contracture [17]. No angular adjustment of the device to wrist contracture was performed in order to gain time as there were many outcome measures in that study.

In the LGMD2C patients assessed in the present study, wrist extension MVC as low as 0.82 N.m could be detected. This is lower than the limit of agreement determined from the results of healthy subjects. However, as the difference between retest and test seems smaller for the weakest strengths (Figure 3), the limit of agreement of weak patients may be different from that of healthy subjects. A larger number of LGMD2C patients would be required to determine the reliability properties of the measurement in this population of weak subjects.

Comparing the patients’ wrist extension torque with the predictive value calculated from the models computed in this study, we quantified the strength deficit. Presentation of the patients’ results relative to predictive values has the advantage of discarding the height confounding factor as it is taken into account in the model. This is of a particular interest in clinical trials with growing children. No relationship between torque and age was observed in this small group of patients. Even if this preliminary observation should be confirmed in a larger patient population, we think this reflects a stabilisation of the wrist extension strength deficit between 16 and 38 years old and not a lack of device sensitivity. Indeed, the sensitivity of the MyoWrist appeared sufficient to detect an inverse correlation of wrist extension torque with age in DMD patients [17]. Moreover, the dynamometer detected in our group of LGMD2C patients a wrist extension torque systematically weaker in the dominant side than in the non-dominant side. The mean difference and SD represented 1.06 ± 0.52 N.m or 17.61 ± 9.13% of the predicted value. First, this indicates that differences as small as ~1 N.m can be discriminated by the device. Second, this suggests that overuse might be deleterious for skeletal muscles of patients with LGMD2C. We found no data in the literature on the effect of exercise on muscle contraction in the specific LGMD2C subtype. Nevertheless, a similar conclusion was obtained for ankle dorsi- and plantarflexors of patients with limb girdle muscle dystrophy as their preferred side was more affected [26], even if the preferred side has probably less impact on the lower limbs than on the upper limbs. Other studies reported, however, increased strength and endurance in wrist flexion and extension [27] and no abnormal creatine kinase level in response to high intensity exercise in patients with LGMD2 [28]. The beneficial or deleterious effect of dystrophic muscle contraction in LGMD2C disease needs to be further investigated.

## Conclusions

The present study precisely describes properties of wrist flexion and extension strength measurement with the MyoWrist. This device provides both wrist flexion and extension strength measurement with the same positioning of the subject. Demonstration of the feasibility of the measurements in healthy or weak children and adults with good reliability guarantees the quality of results for clinical trials. Subjects’ performance can be compared to predicted values computed from normative data collected in healthy children and adults.

Because it is portable and inexpensive, hand held dynamometry is used in strength assessments of the upper limb of patients with neuromuscular disease [29,30]. However, a recent review concluded that because of lack of intra-rater reliability, HHD should not be used for wrist strength evaluation in treatment efficacy studies [31]. Here, we demonstrate that the MyoWrist device which is portable and cheap has good intra-rater reliability probably because of the absence of examiner strength influence. The measurements can be performed in wheelchair-bound patients and if contractures are present, the device allows adjustment of the wrist angle. Therefore, it represents a good alternative to HHD for wrist flexion and extension strength measurement in clinical trials on neuromuscular disorders.

## Abbreviations

LGMD2C: Limb girdle muscle dystrophy type 2 C
MMT: Manual muscle testing
HHD: Hand-held dynamometry
MVC: Maximal voluntary contraction
ICC: Intra-class correlation coefficient
SEM: Standard error of measurement
CVar: Coefficient of variation
SDD: Smallest detectable difference

## References

[1] Mazzone ES, Vasco G, Palermo C, Bianco F, Galluccio C, Ricotti V (2012). A critical review of functional assessment tools for upper limbs in Duchenne muscular dystrophy. Dev Med Child Neurol.

[2] Govoni A, Magri F, Brajkovic S, Zanetta C, Faravelli I, Corti S (2013). Ongoing therapeutic trials and outcome measures for Duchenne muscular dystrophy. Cell Mol Life Sci.

[3] Paternostro-Sluga T, Grim-Stieger M, Posch M, Schuhfried O, Vacariu G, Mittermaier C (2008). Reliability and validity of the Medical Research Council (MRC) scale and a modified scale for testing muscle strength in patients with radial palsy. J Rehabil Med.

[4] Rider LG, Koziol D, Giannini EH, Jain MS, Smith MR, Whitney-Mahoney K (2010). Validation of manual muscle testing and a subset of eight muscles for adult and juvenile idiopathic inflammatory myopathies. Arthritis Care Res (Hoboken).

[5] Ciesla N, Dinglas V, Fan E, Kho M, Kuramoto J, Needham D (2011). Manual muscle testing: a method of measuring extremity muscle strength applied to critically ill patients. J Vis Exp.

[6] Andrews AW, Thomas MW, Bohannon RW (1996). Normative values for isometric muscle force measurements obtained with hand-held dynamometers. Phys Ther.

[7] Vaz DV, Cotta Mancini M, Fonseca ST, Vieira DS, de Melo Pertence AE (2006). Muscle stiffness and strength and their relation to hand function in children with hemiplegic cerebral palsy. Dev Med Child Neurol.

[8] Vanswearingen J (1983). Measuring wrist muscle strength. J Orthop Sports Phys Ther.

[9] Salonikidis K, Amiridis IG, Oxyzoglou N, de Villareal ES, Zafeiridis A, Kellis E (2009). Force variability during isometric wrist flexion in highly skilled and sedentary individuals. Eur J Appl Physiol.

[10] Salonikidis K, Amiridis IG, Oxyzoglou N, Giagazoglou P, Akrivopoulou G (2011). Wrist flexors are steadier than extensors. Int J Sports Med.

[11] Prodoehl J, MacKinnon CD, Comella CL, Corcos DM (2006). Strength deficits in primary focal hand dystonia. Mov Disord.

[12] Alizadehkhaiyat O, Fisher AC, Kemp GJ, Frostick SP (2007). Strength and fatigability of selected muscles in upper limb: assessing muscle imbalance relevant to tennis elbow. J Electromyogr Kinesiol.

[13] Raschner C, Platzer HP, Patterson C, Zeppetzauer M, Del Frari B, Estermann D (2010). An isometric hand tester: quantifying motor function in the hand. J Hand Surg Eur Vol.

[14] Moraux A, Canal A, Ollivier G, Ledoux I, Doppler V, Payan C (2013). Ankle dorsi- and plantar-flexion torques measured by dynamometry in healthy subjects from 5 to 80 years. BMC Musculoskelet Disord.

[15] Kirshner J, Lochmüller H (2011). Sarcoglycanopathies, vol. 101 (3rd series).

[16] Herson S, Hentati F, Rigolet A, Behin A, Romero NB, Leturcq F (2012). A phase I trial of adeno-associated virus serotype 1-gamma-sarcoglycan gene therapy for limb girdle muscular dystrophy type 2C. Brain.

[17] Servais L, Deconinck N, Moraux A, Benali M, Canal A, Van Parys F (2012). Innovative methods to assess upper limb strength and function in non-ambulant Duchenne patients. Neuromuscul Disord.

[18] Beckerman H, Roebroeck ME, Lankhorst GJ, Becher JG, Bezemer PD, Verbeek AL (2001). Smallest real difference, a link between reproducibility and responsiveness. Qual Life Res.

[19] Bialocerkowski A, Grimmer KA (2003). Measurement of isometric wrist muscle strength–a systematic review of starting position and test protocol. Clin Rehabil.

[20] Matsuse H, Iwasa C, Imaishi K, Nago T, Tagawa Y, Kakuma T (2010). Hybrid-training method increases muscle strength and mass in the forearm without adverse effect of hand function in healthy male subjects. Kurume Med J.

[21] Divekar NV, John LR (2013). Neurophysiological, behavioural and perceptual differences between wrist flexion and extension related to sensorimotor monitoring as shown by corticomuscular coherence. Clin Neurophysiol.

[22] Hogrel JY, Decostre V, Alberti C, Canal A, Ollivier G, Josserand E (2012). Stature is an essential predictor of muscle strength in children. BMC Musculoskelet Disord.

[23] Allenbach Y, Benveniste O, Decostre V, Canal A, Eymard B, Herson S (2012). Quadriceps strength is a sensitive marker of disease progression in sporadic inclusion body myositis. Neuromuscul Disord.

[24] Hogrel JY, Allenbach Y, Canal A, Leroux G, Ollivier G, Mariampillai K (2014). Four-year longitudinal study of clinical and functional endpoints in sporadic inclusion body myositis: Implications for therapeutic trials. Neuromuscul Disord.

[25] Romero NB, Braun S, Benveniste O, Leturcq F, Hogrel JY, Morris GE (2004). Phase I study of dystrophin plasmid-based gene therapy in Duchenne/Becker muscular dystrophy. Hum Gene Ther.

[26] Belanger AY, Noel G, Cote C (1991). A comparison of contractile properties in the preferred and nonpreferred leg in a mixed sample of dystrophic patients. Am J Phys Med Rehabil.

[27] Sveen ML, Andersen SP, Ingelsrud LH, Blichter S, Olsen NE, Jonck S (2013). Resistance training in patients with limb-girdle and becker muscular dystrophies. Muscle Nerve.

[28] Andersen SP, Sveen ML, Hansen RS, Madsen KL, Hansen JB, Madsen M (2013). Creatine kinase response to high-intensity aerobic exercise in adult-onset muscular dystrophy. Muscle Nerve.

[29] McDonald CM, Henricson EK, Abresch RT, Florence J, Eagle M, Gappmaier E (2013). The 6-minute walk test and other clinical endpoints in duchenne muscular dystrophy: reliability, concurrent validity, and minimal clinically important differences from a multicenter study. Muscle Nerve.

[30] McDonald CM, Henricson EK, Abresch RT, Florence JM, Eagle M, Gappmaier E (2013). The 6-minute walk test and other endpoints in Duchenne muscular dystrophy: longitudinal natural history observations over 48 weeks from a multicenter study. Muscle Nerve.

[31] Schrama PP, Stenneberg MS, Lucas C, van Trijffel E (2014). Intra-examiner reliability of hand-held dynamometry in the upper extremity: a systematic review. Arch Phys Med Rehabil.

